# Diagnostic assessment of autism in adults – current considerations in neurodevelopmentally informed professional learning with reference to ADOS-2

**DOI:** 10.3389/fpsyt.2023.1258204

**Published:** 2023-10-05

**Authors:** Eleanor Curnow, Izy Utley, Marion Rutherford, Lorna Johnston, Donald Maciver

**Affiliations:** School of Health Sciences, Queen Margaret University, Musselburgh, United Kingdom

**Keywords:** ADOS-2, diagnostic assessment, Scotland (United Kingdom), neurodiversity affirming practice, autism (ASC)

## Abstract

Services for the assessment and diagnosis of autism in adults have been widely criticized and there is an identified need for further research in this field. There is a call for diagnostic services to become more accessible, person-centered, neurodiversity affirming, and respectful. There is a need for workforce development which will increase capacity for diagnostic assessment and support for adults. ADOS-2 is a gold-standard diagnostic assessment tool for autism recommended in clinical guidelines. However, diagnostic procedures such as the ADOS-2 are rooted in the medical model and do not always sit comfortably alongside the neurodiversity paradigm or preferences of the autistic community. Training and educational materials need to account for the differences between these approaches and support clinicians to provide services which meet the needs of the adults they serve. The National Autism Implementation Team worked alongside ADOS-2 training providers to support clinicians in Scotland, to provide effective and respectful diagnostic assessment. The team engaged with clinicians who had attended ADOS training to identify areas of uncertainty or concern. Training materials were developed to support ADOS assessors to incorporate key principles including “nothing about us without us”; “difference not deficit”; “environment first”; “diagnosis matters,” “language and mindsets matter”; and “a neurodevelopmental lens,” to support the provision of neurodiversity affirming assessment practice. The National Autism Implementation Team also provided examples of actions which can be undertaken by clinicians to improve the assessment experience for those seeking a diagnosis. Training materials are based on research evidence, clinical experience, and the needs and wishes of autistic people.

## 1. Introduction

Feedback from neurodivergent people has confirmed that diagnostic assessment offers them significant benefits including personal understanding of being different rather than broken, accessing appropriate support and interventions, finding other people with similar experiences and preferences, and understanding why mental health interventions have been unsuccessful (1, 2). Delayed, missed and misdiagnoses of neurodevelopmental differences, including autism and ADHD, reduce wellbeing, limit access to relevant supports or adjustments, and increase stress and distress (3). Historically, services have focussed on the identification of autistic children but following the development of guidelines for diagnosis of autistic adults (4, 5) rising numbers of adults are seeking diagnostic assessment (6) increasing delays in accessing assessment services (7).

This paper will describe the work of the National Autism Implementation Team (NAIT) who are working alongside other organizations including the Scottish ADOS Consortium and NHS Education for Scotland (NES) to build capacity within the workforce for timely and effective autism diagnostic assessment for adults within developing neurodevelopmental pathways. Pathways are co-designed with autistic people and those with related neurodevelopmental conditions (8).

Particularly, this paper will focus on learning from training events which aimed to support clinicians providing diagnostic assessment for adults. This paper addresses the knowledge gaps of clinicians regarding the use of ADOS-2 with adults in a neurodevelopmentally informed and neurodiversity affirmative practice. It is based on reflections and engagement with multidisciplinary professionals and individuals with lived experience, gathered during training events and NAIT’s community engagement efforts. A framework and accompanying online resources have been developed by NAIT to support this approach. Our work integrates research evidence, clinical experience, and key principles for practice. These principles, which serve as the foundational features of the NAIT program, were developed to communicate intentions and values. This paper will demonstrate how these principles are being applied to adult assessment and the use of the ADOS-2. By highlighting the practical implementation of these principles in the context of adult assessment, we provide insights into their effectiveness and applicability in improving the assessment process for neurodevelopmental conditions in adults.

Autistic adults and professionals have expressed dissatisfaction with the assessment and diagnosis process in the United Kingdom (9, 10) and have identified this as a priority for further research (11). Particular challenges facing autism identification in adulthood include: the paucity of adult-specific screening and diagnostic tools; vague and inconsistent routes for accessing diagnosis; possible reduction of outward symptom severity and visibility of co-occurring conditions later in life; poor recall of early-life developmental history; cultural factors that may mask autistic signs; the absence of key professionals; the tendency of professionals to focus on negative rather than positive aspects of autism; lack of rapport with professionals; tensions between professionals and autistic people regarding who is the expert; lack of clarity surrounding the diagnostic process; inappropriate spaces for assessment to take place; and limited experience and training in adult autism of many professionals (2, 7, 9, 10, 12–14). Research has found evidence that some autistic people felt the diagnostic experience was not age or gender appropriate (10, 15), did not consider sensory preferences, that assessment took place in an inappropriate environment, information provided before the assessment was limited (10) and there was inadequate post-diagnosis or long-term support (13). Some people have even found receiving an autism diagnosis to be a traumatic experience (16).

Traditionally, the medical model has focused on diagnostic assessment to identify disorders using defined clusters of symptoms to facilitate communication and shared understanding, and as a means of accessing appropriate support (1, 17, 18). DSM-5 includes five criteria to define who has or does not have autism, these include; persistent deficits in social communication and social interaction; restricted, repetitive patterns of behavior, interests or activities; symptoms were present in the early developmental period and clinically significant impairment in functioning that are not better explained by another condition (19). These criteria have been criticized as being open to interpretation regarding what does significant impairment mean, and does this impairment arise from the individual or their environment (18). Additionally, autistic people may employ behaviors including masking or camouflaging to avoid appearing to be functionally impaired which can lead to depression, anxiety, and suicidality (20, 21).

In contrast to disorder-based frameworks, the neurodiversity paradigm moves the focus of autism interventions towards an understanding of how the physical and social environment constrains and limits neurodivergent people (22). Becoming neurodiversity affirming requires services to accommodate naturally occurring variability and be centered around the perspectives and experiences of neurodivergent people (12). Such services promote autism acceptance and aim to improve quality of life and well-being while respecting and preserving autistic ways of being and, importantly, are provided at the request and with the consent of the autistic people in question (23, 24).

To provide respectful, accessible, and patient-centered services, there is a need for workforce development (9). NAIT has been working with other national organizations to facilitate training for clinical teams to increase capacity for high quality autism diagnosis and support for adults, using research evidence, clinical experience and neurodiversity affirming principles. Training materials, course content and the diagnostic process are intended to reflect the preferences of the autistic community (9, 10).

This paper will focus particularly on one diagnostic tool – ADOS-2 (25, 26) which is considered a gold standard diagnostic assessment tool for autism recommended in NICE (4) guidelines alongside the Autism Diagnostic Interview – Revised (ADI-R) (27). ADOS-2 is a semi-structured standardized clinician observation measure. It is recommended that ADOS-2 should not be used alone but in combination with broader clinical assessment to determine whether an autism diagnosis is appropriate (28).

However, understanding about autistic thinking and perception has progressed since the ADOS-2 was designed. ADOS-2 has been criticized for its use of deficit focussed language, viewing autism through a medical model lens, and for processes which can leave the person being assessed feeling as if they have been manipulated (29). There is therefore a need to provide clinicians with support regarding how and when they can use this tool to inform the diagnostic process whilst maintaining the wellbeing, confidence and trust of the person being assessed. Additionally, concepts such as masking (30) and alexithymia (31) may not be explicitly considered in current diagnostic tools including ADOS-2.

## 2. Context

This paper describes the work of NAIT to attempt to provide clinicians with evidence-based information which will support the provision of respectful, accessible and patient-centered autism diagnostic assessment for adults in line with current guidelines (4, 5).

This work is taking place nationally across Scotland. The prevalence of autism in Scotland is not known (32). Census data indicates that 6,649 (0.2%) of adults over the age of 25 in Scotland are autistic (33). As prevalence of autism for children aged 0–15 years in the same year was estimated at 1.9%, and 1.2% for young people aged 16–24, there is an indication that there may be a significant proportion of autistic adults who remain unidentified (34). Lack of diagnostic services for adults in Scotland who may be autistic has been identified as a challenge, alongside a need for training in standardized assessment tools to aid diagnostic accuracy (7). Community Mental Health Teams (CMHT) have been responsible for providing diagnostic assessment but their thresholds for access mean that referrals for people seeking diagnosis and support may be rejected (2). Additionally, CMHTs often focussed on providing pharmacological solutions and psychological therapies which may not be neurodevelopmentally informed (2). Adaptations to existing services and mindsets are required alongside the creation of new services, and the development of a workforce with the skills to meet the needs of adults with neurodevelopmental conditions (2). To meet professional learning needs in 2022–2023, training in ADOS-2 was provided by the Scottish ADOS Consortium, the official provider of ADOS training in Scotland in conjunction with NHS Education for Scotland (NES), for staff working within adult autism diagnostic assessment teams. So far 158 clinicians have received ADOS training in Scotland during 2022-2023, thirty of these were working in adult services and seven in whole lifespan services, whilst the remainder work in services for children.

## 3. Detail to understand key programmatic elements

The National Autism Implementation Team (NAIT) is funded by the Scottish Government to bridge the evidence and policy to practice gap and improve health and education provision for neurodivergent people (8, 35, 36). The wider NAIT program aims to update professional learning and create a neurodevelopmentally informed workforce through various mechanisms (2, 8). These mechanisms included engaging with national strategic groups and government officials, providing evidence-informed information, promoting universal inclusive practice, building consensus on acceptable language use, modeling expected behaviors, adopting a national focus, and providing a systematic framework for sustainable practice through high quality professional learning materials. Activities and interventions involved establishing a multidisciplinary team, consulting with adults with lived experience, meeting with government officials, developing resources and practice guides, offering professional learning activities and training, providing individual support meetings, creating evidence-based practice guides, developing neurodevelopmental pathway frameworks, and establishing assessment frameworks for adults focusing on ADHD and autism. These measures aimed to facilitate the application of knowledge, enhance skills, and provide guidance to professionals, ultimately achieving the goals of the NAIT program in improving assessment, diagnosis, and interventions for neurodevelopmental conditions.

Specific to adult provision, NAIT supports four ‘pathfinder’ innovation sites across Scotland, to develop sustainable services which will provide assessment and services to adults seeking autism diagnosis and support by providing training in diagnostic skills and expertise in establishing services competent to identify autism in adults (37). These services are intended to reflect values embedded within the neurodiversity paradigm, social models of disability and person-centered practice (23, 38).

Prior to inception of the innovation sites, assessment and diagnosis of autism and other neurodevelopmental conditions were undertaken by community mental health teams (CMHT). However, limited resources meant that individuals seeking autism assessment often found there was either no local service available or they were faced with long waiting times (2). Additionally, interventions provided by CMHT’s often focus on pharmacological and psychological therapies which were not always neurodevelopmentally informed or appropriate for autistic people (8). Clinicians reported that change was difficult as they did not know which resources to trust, and which practices were safe or effective (8). NAIT collaborated with key partners to build capacity across professional groups in neurodevelopmental teams to undertake diagnostic assessment within a neurodiversity affirming framework.

There was a recognized need from people with lived experience, government officials and clinicians for further training in the use of diagnostic assessment tools including ADOS-2 to meet increased levels of demand in adult services (35, 36). ADOS-2 is used in all children’s services but is less widely used within adult services in Scotland (7).

To meet this demand, workforce development including ADOS training was targeted at a broader range of professionals than were previously involved in assessment, diagnosis and support services (2). Based on engagement after these training events, participants were clearly actively engaged in a valuable learning process, generating important insights. Questions raised at these events are summarized in Table 1.

**Table 1:** Frequently asked questions (FAQs).

	Question
1.	When is ADOS indicated as part of a diagnostic assessment?
2,	Can you use ADOS with adults with and without Intellectual disability?
3.	Can you use ADOS-2 for adults with co-occurring neurodevelopmental and mental health conditions?
4.	What is an ADOS informed assessment?
5.	Can you use ADOS remotely or only face-to-face?
6.	What ADOS materials can be adapted to make it more appropriate for adults?
7.	How do you interpret the scores? Are these standardized for adults?
8.	How do you report findings?
9.	Is ADOS compliant with a neurodiversity informed perspective?
10.	Does ADOS work equally well for all genders and ethnic/cultural backgrounds?

To share and disseminate the knowledge gained from these events and advance neurodevelopmental assessment practices to support individuals in need, a resource was created. This resource, available online, complements this paper and provides practical guidance for clinicians. This ‘NAIT guide to using ADOS with adults’ promotes the adoption of a neurodevelopmentally informed practice by demonstrating appropriate language usage, providing illustrative examples of how to apply ADOS-2 in an informed manner with adults, and clinical indicators to assist in the identification of complexities in diagnosis that may require further investigation or expertise. The content of the documentation was informed by a rapid review of published literature and aligned with NAIT’s principles of neurodevelopmentally informed practice. It underwent thorough review and improvement by members of the NAIT team and the Scottish ADOS consortium. Clinicians and professionals can access the guide in Supplementary materials. Together, the paper and the documentation aim to support clinicians in implementing neurodevelopmentally informed practices and facilitating accurate and meaningful assessments for individuals with neurodevelopmental conditions.

In addition to the engagement events and the development of online resources, this paper goes beyond their provision by offering further reflection, discussion, and debate on the application of the NAIT principles. Its purpose is to stimulate a robust and constructive dialog among professionals, fostering an environment of ongoing learning and improvement in the field of neurodevelopmental assessment, with a strong focus on listening to and including neurodivergent people as partners.

### 3.1. Key learning on adult assessment and NAIT key principles

It is crucial to adopt innovative approaches that foster collaboration between autistic individuals and their allies. While such practices may not be well defined or supported by clinical guidelines, the key principles that underpin the work of NAIT provide valuable insights and can serve as a foundation. These principles aim to establish more acceptable, timely, and effective assessment pathways. The principles guiding the work of NAIT are: “Nothing about us without us,” prioritizing co-production with autistic and neurodivergent individuals; “Difference not deficit,” recognizing and embracing neurodiversity; “Environment first,” creating supportive environments; “Diagnosis Matters,” emphasizing the significance of accurate diagnosis; “Language Matters,” promoting respectful and inclusive language; and a “Neurodevelopmental lens,” considering co-occurring conditions. This paper will explore these principles and their implications for advancing assessment practices and shaping future guidelines, ensuring the meaningful involvement and well-being of autistic and neurodivergent individuals.

#### 3.1.1. Nothing about us without us

The principle of “nothing about us without us” underscores the critical importance of co-production with autistic and other neurodivergent individuals in all aspects of decision-making and practice development. It emphasizes the need to ensure that power is shared, and that those with direct experience of being neurodivergent have an active and meaningful role in shaping policies, services, and research agendas. Moving away from paternalistic medical model approaches, this principle recognizes the expertise and lived experiences of autistic and neurodivergent individuals as valuable contributions to shaping more inclusive and effective practices. By embracing co-production, we can foster a more collaborative and empowering approach that respects the rights and agency of individuals and promotes their active participation in decision-making processes that directly impact their lives.

This principle is upheld during the assessment process by recognizing the individual as the expert, shifting power away from clinicians towards neurodivergent people and communities. As already stated, autistic adults report that diagnosis can enhance self-knowledge, identity and community (39), but diagnostic labels and support services have remained under the control of clinicians. In recognition of the autistic adult as expert, there is a move towards valuing self-identification of being autistic or having related needs which elicit provision of appropriate accommodations and supports (39). Neurodiversity affirming practice envisions a future where standardized assessments rooted in the medical model may be disregarded in adult assessment altogether. Instead, the focus will be on exploring assessment methods which align with neurodiversity principles, valuing individuals’ unique neurodevelopmental profiles (40). There may also be a future where diagnosis is no longer required and there is a more direct path to self-understanding and the provision of required support (39).

An important aspect of the diagnostic assessment process is that it is where possible, the choice of the individual. To make an informed decision about whether to undergo diagnostic assessment, the person requires an understanding of the process and the possible assessment outcomes. In situations where the individual concerned does not have capacity to make this choice the decision to undertake diagnostic assessment can be made by an appropriate representative or guardian. Suggestions regarding the introduction of ADOS-2 to adults seeking diagnostic assessment or their representative, include providing information about the nature of the assessment in advance to allow people to provide informed consent regarding undertaking diagnostic assessment and to prepare themselves for the process. This part of the process can build trust between the assessor and person being assessed which will enhance the diagnostic experience and encourage the person being assessed to share details of their personal history (1). Trust between the diagnostic professional and the person undergoing assessment is important to achieve satisfaction with the assessment process (38). This can also promote a sense of shared responsibility between the clinician and person seeking assessment. This relationship is important and people can be more accepting of assessment measures if the clinician is able to interpret results rather than using them as a blunt instrument (1).

#### 3.1.2. Difference not deficit

Neurodevelopmentally informed practitioners face the challenge of navigating between the deficit-focussed language used in current diagnostic criteria and assessment tools, and the preferences of neurodivergent individuals for updated, respectful language and mindsets. Recognizing that autism and ADHD are differences rather than deficits, these practitioners strive to adopt a more inclusive and empowering approach that respects the unique strengths and abilities of individuals with neurodevelopmental conditions. This does not mean that tools are no longer valid, but rather that some translation is required in how clinicians interpret and describe or report what they see in assessments (18, 41). It is anticipated that this approach will also influence clinicians to move away from interventions which try to “fix” people towards facilitation of environmental and participation focussed support.

#### 3.1.3. Environment first

A neurodevelopmentally informed assessment process, understands the influence of the range of physical and social environments experienced rather than seeing an ‘in- person’ deficit. In an optimal environment the person may not experience or demonstrate the negative manifestations of communication, sensory or thinking style differences – but they are still autistic. In a sub-optimal environment, individuals may experience significant distress but may or may not mask or express this in ways described in diagnostic criteria (42, 43). The diagnostic process can be a traumatic event for some adults (16). Although, consideration of the environment and the relationship between practitioner and autistic adult can help support a trauma informed approach, individuals should feel safe within the assessment space. ADOS-2 can take place within a supportive environment where communication and sensory preferences are considered and supported.

#### 3.1.4. Diagnosis matters

Autistic adults and those with other neurodevelopmental conditions send a strong message that diagnosis matters. Timely, effective assessment and diagnosis, can support individuals to: develop their own identity and a renewed understanding of themselves as different and not broken (1, 44); identify peers with shared experiences and a voice within a neurodivergent community; locate neurodevelopmentally informed information about autism and what that means for them; access support and information for their wider family, community, professionals who support them and educators or employers; and can open up conversations and reflections on prioritizing supports and adjustments that support meaningful participation in naturally occurring environments (1, 44).

Previous research has identified inadequate information on the meaning of an autism diagnosis, or accessing relevant support as a source of dissatisfaction following diagnostic assessment (9). Clinicians can empower adults through increasing their understanding of the outcome of any assessment procedure and focussing on positive environmental changes which will enhance their wellbeing and quality of life (45). Engendering positive emotions about an autism diagnosis can increase the confidence of the individual (45). Providing appropriate support before, during and after diagnosis can assist in increasing the acceptability of diagnosis and emphasizing individual strengths. By focussing on the strengths of the individual within the assessment report the assessor can promote a neurodiversity affirming approach with other clinicians who may be associated with their care.

For some individuals, their preference is not to take part in diagnostic processes or not to identify with a particular diagnostic description. They are absolutely within their rights to make this decision and also to change their minds over time. Decisions around disclosure of support needs or diagnosis are complex. Receiving a diagnosis of being autistic, does not oblige individuals to disclose their autistic identity (46).

#### 3.1.5. Language matters

The guide promotes the use of strengths focussed language during assessment and in diagnostic reports, by modeling preferred and inclusive language and terminology (47). Neurodiversity affirming recommendations for practice include detailing the actions and responses of the individual during the assessment, rather than reporting the ADOS-2 score. Instead of categorizing the individual, this description may facilitate better understanding of the challenges facing the person and how these may be relieved through adaptation of their environment (22). This moves away from a focus on providing a clinical diagnosis to identifying preferences or aspects of the environment which present challenges for the individual (39) and which can be adapted (45). Focussing on the strengths of the individual will also prevent ADOS scores being used to determine access to clinical services (48), by describing services or environmental adaptations which may meet identified needs.

ADOS-2 is helpful, as a standardized assessment, in the sense that the process requires the creation of an optimal communication environment (albeit within a clinical setting) with assessment taking explicit account of how the actions of the examiner influence responses or how the opportunities and experiences are affected by that environment. However, it must be acknowledged that this brief observation should be supported by detailed consideration of the developmental history and experiences of the individual being assessed (48).

#### 3.1.6. Neurodevelopmental lens

It is fundamental to expect co-occurrence. In mental health, historically the outcome of diagnostic assessment is reported (e.g., anxiety or depression) often with reference to family environment and trauma but not necessarily with an understanding of neurotype or the influence of aspects of naturally occurring environments.

Co-occurrence is normal and not the exception (49). There is a need to understand autism in the context of other psychiatric and neurodevelopmental conditions (e.g., ADHD, FASD, DCD, DLD, ID) (49). Accurate diagnosis combined with detailed consideration of an individual’s cognitive, language and adaptive behavior skills will direct people to interventions and support which are appropriate for their needs. Historically, clinical guidelines have considered single conditions, but the high prevalence of co-occurrence indicates that neurodevelopmental pathways should consider individual profiles and needs. Diagnostic assessment tools can assist clinicians in identifying clinically relevant information to support diagnosis but have not often been validated on sample populations which present with specified co-occurring conditions (48).

Internationally, there is a new awareness of the range of ways autism is experienced by individuals with different neurotypes. These can be better understood through identifying autism in the context of co-occurrence of other neurodevelopmental conditions or resultant mental health conditions. Under ascertainment of ADHD in adults is a significant issue in Scotland (2), and there is a need to undertake autism assessment in the context of co-occurring disability, or to distinguish between diagnoses with overlapping presentations. Being trained in using ADOS-2 supports the professional team to have a neurodevelopmentally informed lens on diagnostic assessment, even if this tool is not used in every assessment.

It is estimated that 28–44% of autistic people have ADHD (50), 10–54% experience anxiety or depression, and there is a recognized overlap in the symptoms of autism and borderline personality disorder (51). The overlapping of characteristics associated with both autism and mental health diagnoses such as personality disorder, depression, or anxiety, can lead to misdiagnosis, missed diagnosis or diagnostic uncertainty (50, 51). There is some evidence of the need to re-think diagnostic mindsets and labels, in particular historical mental health diagnoses, where neurodevelopmental differences have not been recognized (52) or where diagnostic criteria have changed over time. Factors which have contributed to this problem may include clinicians lacking confidence to diagnose autism in the presence of other conditions such as intellectual disability. This means that undertaking any autism assessment, including ADOS-2, requires an understanding of how observations might be affected by symptoms associated with mental ill-health or other neurodivergence. Instead of focussing on specific symptoms, clinicians must consider wider context through detailed clinical history, discussion and observation (53). NAIT therefore sought to provide information for clinicians on ADOS-2 results, which places a neurodevelopmental lens on the possible co-occurrence of a mental health diagnosis where autism assessment outcomes warrant further investigation (53, 54).

## 4. Discussion

Clinical guidelines (4, 5) advise diagnosticians on current standards for differential diagnosis of neurodevelopmental conditions in adults, using ICD-11 or DSM 5, through formulation based on evidence gathered in a range of ways. This should include early developmental, medical, and family history; information about past and current experiences in different contexts (e.g., home, education, work); and direct observation of and interaction with the individual being assessed (this may be online or face to face). Use of standardized assessment tools such as ADOS-2 is not essential but can be helpful and is recommended in some cases (55), and can increase clinician competence (56). Diagnosis should never be based on the outcome of one assessment tool or one part of the process alone. The life experiences of the autistic adult should be listened to and validated throughout the assessment process (40). However, evidence suggests that the experience of clinical diagnostic assessment is not always positive (1, 9, 13).

Diagnostic rates of 80–90% are reported in adult services in Scotland and highlight that when someone has lived their life into adulthood and thinks that they might be autistic, this is very often the case and ADOS-2 may not be necessary in making a full assessment (2). Often the more difficult scenario is reaching the decision that the person is not autistic when they have described challenges they experience in different environments, which they have come to understand as occurring because they are autistic. There is a need for professional teams with the right skills, assessment processes and tools which enable differential diagnosis, and identification of other neurodevelopmental or mental health conditions and identification of support needs and profile, rather than a single decision that the individual is not autistic (48, 53).

To improve satisfaction with the diagnostic experience, NAIT developed a range of resources to support clinicians to facilitate good quality assessments for autistic adults in a neurodevelopmentally affirmative way. This work is a key example of mechanisms used to support key NAIT outcomes and is intended to be accessible, concise, engaging and evidence-informed, and consistent with the NAIT principles of neurodevelopmentally informed practice described above. Recent evaluation of the NAIT program found that stakeholders felt materials published by NAIT were likely to be used in practice as they were evidence-informed, succinct and thoughtfully presented (8). The key messages and underlying principles support autonomy.

The NAIT guide to using ADOS with adults represents NAIT engagement with clinicians involved in the provision of diagnostic assessment and is based on questions raised following ADOS-2 training sessions. Sharing these questions with national groups such as The Scottish ADOS consortium facilitates understanding of issues facing clinicians, by people at more senior or strategic levels. It is also a means to share clinical knowledge and experience.

The “What this means in Practice” sections model expected behaviors – providing practical examples of neurodiversity affirming practice, using inclusive, preferred language and terminology (8). These examples support the transfer of values from theory to practice through the provision of examples and practical advice for the ‘translation’ of evidence.

Seeking diagnosis or identification of autism can be difficult and time consuming (1). This guide is an example of NAIT activity to provide clinicians with support to facilitate optimal assessment services. It is intended to reduce aspects of diagnostic assessment that have previously been found to make people uncomfortable such as limited transparency regarding the assessment process, age-appropriateness of activities included in the assessment, and poor rapport with the assessor (10). The guide provides advice based on research evidence and clinical experience and is intended to increase transparency surrounding ADOS-2 assessment. This summary of research evidence relevant to practice is intended to support health professionals develop confidence in ADOS assessment and illustrate language and practices which can bridge the gap between traditional deficit-based diagnosis and neurodiversity affirming practices. It may also support good practice more generally. This can assist the clinician to frame diagnosis in a positive way for the autistic adult (9). The guide models the use of current inclusive and preferred language and terminology. Clinician feedback on the guide praised the interpretation of available evidence that could be used to improve practice.

People accessing ADOS-2 assessment need to know about the nature of the assessment, what it is for, and available alternatives to allow them to make an informed decision whether to participate in the assessment process or not. People should be able to access this assessment in a supportive environment and in a way that their communication and sensory preferences are respected. The ADOS-2 assessment should never be performed in a way which causes the person to experience trauma during assessment.

Clinicians have to assist the person seeking diagnostic assessment to consider what an autism diagnosis may mean to them and how it may or may not assist them in accessing required support (9). There is a need for clinicians to be able to explain the steps of the diagnostic process and to ensure the person receives information and support whatever the outcome. Increased awareness of co-occurring conditions or conditions with similar presentations may assist the clinician to access alternative diagnosis and appropriate supports for the person.

This guide will not change practice on its own but is intended to provide evidence-based information which supports the adoption of the principles underlying the neurodevelopmental paradigm throughout all aspects of their service. A recent service review concluded that NAIT was effective in supporting the implementation of new working practices using a range of activities and resources (8). Neurodevelopmentally informed practice is everyone’s business and feeling informed about how ADOS-2 accords with this approach is an essential element of professional learning. It is anticipated that this NAIT information guide will provide support to clinicians choosing to use the ADOS-2 assessment within a neurodiversity informed approach.

### Acknowledgment of any conceptual or methodological constraints

This manuscript is based upon published literature, and the knowledge and clinical experience of members of the Scottish ADOS Consortium, NES, and National Autism Implementation Team in Scotland. The applicability of this information to services outside the Scottish context is not known. The impact of the ‘NAIT guide to using ADOS with adults’ has not been evaluated with people undergoing diagnostic assessment for autism.

## Data availability statement

The original contributions presented in the study are included in the article/Supplementary material, further inquiries can be directed to the corresponding author.

## Author contributions

EC: Investigation, Writing – original draft, Writing – review & editing. IU: Writing – review & editing. MR: Conceptualization, Writing – review & editing. LJ: Writing – review & editing. DM: Writing – review & editing.
