# Supplementary material for: Diagnostic assessment of autism in adults – current considerations in neurodevelopmentally informed professional learning with reference to ADOS-2
Source: Front Psychiatry. 2023 Oct 5;14:1258204. doi: 10.3389/fpsyt.2023.1258204 (PMC10585137; doi:10.3389/fpsyt.2023.1258204)
Supplement: Supplementary file 1 [file Data_Sheet_1.pdf]

NAIT guide to using ADOS with Adults

There are many tools and approaches which can be used to gather information for assessment and diagnosis of autism and other neurodevelopmental conditions. The Autism Diagnostic Observation Schedule (ADOS-2) is only one tool which can be used. It is not intended to be used in all assessments or as a stand-alone diagnostic measure. This guidance was developed for professionals, following questions raised by those attending ADOS-2 training.

The Autism Diagnostic Observation Schedule, ADOS -2

ADOS-2 (Lord et al., 2012) is one of the gold standard formal diagnostic assessments for Autism Spectrum Disorder (ASD) alongside Autism Diagnostic Interview – Revised (ADI-R) (Ashwood et al., 2016). The ADOS-2 algorithm is linked to ICD-10 diagnostic criteria.

ADOS-2 is a semi-structured standardised clinician observation measure designed to examine impairments in social communication and the presence of restricted and repetitive behaviours. There is a choice of four modules, with the module selected based on the individual's communication stage and age.

ADOS-2 should not be used alone but in combination with broader clinical assessments to determine whether an autism diagnosis is appropriate (Taylor et al., 2017).

ADOS-2 is used in all children's services but has not been widely used within adult services in Scotland (Rutherford et al., 2016). This guide is intended to address some of the questions that arise in relation to its use with adults.

More information on ADOS-2 and training in Scotland can be found here https://www.ed.ac.uk/lifelong-learning/ados

Frequently asked questions:

1. When is ADOS indicated as part of a diagnostic assessment?

- NICE advice states that ADOS-2 is one of the assessment tools which may aid more complex diagnosis and assessment for adults alongside other aspects of a comprehensive assessment.
- Guidance on consideration of complexity is here.
- ADOS-2 is deemed to be part of the gold-standard for diagnostic evaluation together with a standardised interview (ADI-R) and a differential diagnosis examination by an experienced clinician (Kamp-Becker et al., 2018). It is not a requirement for a robust diagnosis.
- ADOS Module 4 is for people with fluent speech. It has good sensitivity and specificity, and good diagnostic utility when it is used in combination with other sources of diagnostic information and operated by experienced examiners (Langmann et al., 2017; Medda et al., 2019).

What this means in practice:

- ADOS-2 is a useful tool which can assist diagnostic assessment.
- It may be most useful in complex cases such as where the person experiences co-occurring conditions; there is a difference of opinion between the individual being assessed, informants or different clinicians; or the person presents with a complex case history.

© NAIT and the Scottish ADOS Consortium 2022

2. Can you use ADOS with adults with and without Intellectual Disability?

For adults without intellectual disability

- The accuracy of Module 4 has been confirmed.
- Accuracy of Module 4 is greater than ADI-R (Fusar-Poli et al., 2017). Although, the diagnostic accuracy of ADOS may be reduced for individuals with higher intellectual abilities (Langmann et al., 2017).

For adults with intellectual disability

- ADOS-2 was found to be a valuable diagnostic tool for adults, although over-inclusive in this population. It correctly identified 100% of autistic people (sensitivity = 100%). However, it only identified 45% of people who were not autistic (specificity = 45%) (Sappok et al., 2013).
- Limitations in the ADOS for this population include some materials within Modules 1 and 2 are designed for use with young children and may therefore be inappropriate for adults. Replacement items are permitted.
- One study found that ADOS-2 could reliably identify autism in deaf adults with intellectual disability (Holzinger et al., 2022).

What this means in practice:

- Yes, ADOS-2 can be used with adults without intellectual disability.
- ADOS-2 can also be used with adults with intellectual disability who have language and cognitive levels that allow them to participate fully in the assessment process through use of relevant modules.
- ADOS-2 should not be used as the only evidence that an individual meets diagnostic criteria.

3. Can you use ADOS 2 for adults with co-occurring Neurodevelopmental and Mental Health conditions?

There is evidence that 33% of autistic people in Scotland have a co-occurring long-term mental health condition (Rydzewska et al., 2018) and autism is under-diagnosed in adults involved with mental health services. Here is a summary of available research regarding the possible interaction of mental health conditions and ADOS assessment. Other mental health conditions did not feature in the published research discovered in our literature search. However, possible interactions should be considered during the assessment process, for example medication or some mental health conditions could also affect aspects being observed, engagement and responses. Research reported:

Broader autism phenotype

Reported research findings indicate that ADOS-2 Module 4 can be used effectively to distinguish between autism and neurotypical profiles but could not as easily distinguish between autism and the (outdated) term referring to people with the broader autism phenotype who do not meet all autism diagnostic criteria - PDD-NOS (Adamou et al., 2021).

Mental health problems

It is possible that some mental health problems may inflate ADOS scores, and it should therefore be used in conjunction with other assessment tools such as a developmental assessment (Brugha et al., 2020; Stadnick et al., 2015). This finding supports the practice of offering earlier stage assessment to individuals outside engagement in secondary care mental health services. This finding does not support the practice of using thresholds of distress and severity of impairment to reject referrals for people with 'milder' presentations.

Schizophrenia

There is convergence of schizophrenia negative symptoms and autistic phenotype, and many patients appear to (and may) fulfil criteria for both schizophrenia and autism (Kästner et al., 2015). Prevalence of schizophrenia has been reported as significantly higher in autistic people (OR=3.55), and

© NAIT and the Scottish ADOS Consortium 2022

prevalence of autism in people with schizophrenia ranged from 3.4% to 52% (Zheng et al., 2018). However, this may be because ADOS alone does not sufficiently distinguish between autism and schizophrenia (Maddox et al., 2017).

Schizophrenia patients predominantly experiencing negative symptoms obtain high scores on ADOS (Bastiaansen et al., 2011; de Bildt et al., 2016; Hus & Lord, 2014). Negative symptoms in schizophrenia include reduced social-emotional reciprocity, blunted affect, reduced non-verbal communication, apathy, reduced affect sharing, and reduced social overture and response (Kästner et al., 2015).

Disorder specific positive symptomatology differentiated autistic and schizophrenic groups more effectively. People with schizophrenia demonstrated higher positive symptoms related to psychosis (e.g., delusions and hallucinations), whereas those who were autistic demonstrated higher positive symptoms associated with autism including inappropriate overtures, abnormalities in language and speech, restricted interests, and repetitive behaviours. Few positive autism signs were noted in schizophrenia patients (Trevisan et al., 2020).

ADHD
20% of people with ADHD met scores for ASD classification on ADOS-2. ADOS-2 has been used to identify autism in adults with ADHD not clinically diagnosed with ASD (Hayashi et al., 2022). This population showed high scores on Social Affect domain (SA) of ADOS-2 compared to scores in other domains.

Personality disorder
Personality disorder is a recognised condition for which there are treatments available and good outcomes. However, personality disorder has been described as a controversial diagnosis with many autistic people reporting being misdiagnosed. The personality disorder label was wrongly attributed by professionals who did not have adequate autism knowledge.

Research states that people with a diagnosis of personality disorder may be more likely to receive a false-positive autism diagnosis on ADOS. Individuals with personality disorder are described as more likely to exhibit deficits in the Social Affect (SA) and Repetitive and Restricted Behaviours (RRB) domains which may reduce the accuracy of ADOS assessment-based diagnosis. People with personality disorder are reported to exhibit differences associated with theory of mind/ empathy, comprehensive and severe problems in social relations, communications, self-perception, preference for remaining solitary, obsessive-compulsive behaviour and idiosyncrasies (Langmann et al., 2017).

Eating disorder
Previous research has found that up to 53% of people with acute anorexia nervosa scored above the ADOS-2 cut off for a diagnosis of autism (Sedgewick et al., 2019; Westwood & Tchanturia, 2017). In addition to eating-related symptoms, social-emotional and cognitive difficulties play an important role in the development and progression of anorexia nervosa. Severe malnutrition is also known to impact social and cognitive functioning, including social withdrawal, difficulties concentrating and apathy (Sedgewick et al., 2019).

ARFID
There are also similarities in the eating patterns of autistic people and people with Avoidant/ Restrictive Food Intake Disorder (ARFID). Further information is available:
ARFID - Beat (beateatingdisorders.org.uk)
ARFID & AUTISM | ARFID Awareness UK

What this mean in practice:

- In the ADOS, the examiner is seeking to create optimal communication conditions and periods of extreme physical or mental ill-health may not be the optimal time to undertake ADOS. If it is used, the reporting should reflect awareness of this.
- Where the score on one domain of the ADOS is out of sync with scores from other domains, this may indicate the presence of a co-occurring condition. The social communication difficulties measured by ADOS-2 are not unique to autistic people.
- It should also be noted that undiagnosed autism or misdiagnoses may be common in psychiatric hospitals and outpatient programmes.
- ADOS-2 was not designed to be used as a stand-alone diagnostic measure. As mental health conditions may inflate ADOS scores, ADOS should be used in conjunction with developmental history information and clinical assessment to maximise diagnostic accuracy. The impact of the co-occurring mental health condition on ADOS outcomes should be considered.
- Presence of mental health conditions should not exclude autism diagnosis.
- Assessment findings should be discussed with other people involved in the care of the individual, and professionals with relevant expertise before making a diagnosis.

4. What is an ADOS informed assessment?

This term is sometimes used to describe an assessment

a) When the assessor is not using ADOS-2 materials but is mindful of key areas of observation and practice within ADOS-2. For example, making notes about number of presses and opportunities to contextualise observed or non-observed signs.

b) Which uses ADOS –2 materials but where circumstances mean that the administration of the assessment varied from the standardised procedure. Examples include online ADOS assessment, or assessments adapted for people with disability who are unable to participate in the assessment in its standardised form.

What this means in practice:

- Being ADOS-2 trained and experienced can increase clinical assessment skills and judgement in a range of assessment processes.
- In some circumstances it may not be possible to adhere to ADOS-2 standardised procedures exactly as described in the manual. In such circumstances the assessor must take care to document any deviations from the standardised procedure. This may include details such as the number of presses or opportunities offered to the person being assessed, that the assessment took place online, or that alternative materials were used to increase age-appropriateness or accessibility.
- Any final report should also describe the circumstances of the assessment and indicate the impact this may have had on the assessment outcome.

5. Can you use ADOS remotely or only face to face?

- One study (N=14) examined the feasibility, usability, and reliability of administering the ADOS Module 4 remotely (Schutte et al., 2015).
- More participants stated they preferred face-to-face administration (n=5), to the remote system (n=2).
- Facilitating eye contact remotely was challenging and managed using a teleprompter.
- Agreement was low for the following items: “asks for information”, “empathy/ comment on others’ emotions”, “hand and finger and other complex mannerisms”.

- ADOS-2 administrators found that in some cases they could make observations face-to-face that were not possible during remote administration. The researchers emphasise a need for further research on the remote delivery of ADOS -2 assessment (Schutte et al., 2015).

What this means in practice:

- An adapted version of ADOS-2 may be administered online. The assessor should follow guidance for ADOS-2 informed assessment and describe the method of administration in any report of the assessment including details of the impact this may have had on the outcome of the assessment.
- Some adults may have a preference for remote assessment rather than face to face.

6. What ADOS materials can be adapted to make it more appropriate for adults?

- Module 4 is designed for adults but note that some activities are optional if these are thought to be inappropriate for a particular individual.
- Adapted Module 1 and Module 2, were designed for use with minimally verbal adolescents and adults (Bal et al., 2020). The validity of the adapted version was supported for minimally verbal adolescents and adults with nonverbal mental ages of at least 18 months although further validation for older adults is required.
- Adaptations made to Pre-Linguistic ADOS were used to make ADOS tasks and materials more age-appropriate for adults. The Make-Believe Play task was removed.

What this means in practice:

- The ADOS-2 assessor should attempt to offer age and stage-appropriate assessment. It should be recognised that the ADOS-2 may not be the best assessment tool to use for some adults based on their cognitive or language skills.
- Some tasks within the ADOS-2 assessment are optional allowing the assessor to tailor the assessment to individual need.

7. How do you interpret the scores? Are these standardised for adults?

- The accuracy of ADOS-2 scoring depends on the experience of the coder, as well as on characteristics of the cases and the quality of the administration of the ADOS (Kamp-Becker et al., 2018).
- Scores over the 7+ threshold on the ADOS –2 can be recommended for subthreshold community cases and 10+ can be recommended for definite community cases of ASD (Brugha et al., 2012).
- Modified guidelines which include operational definitions for each area of observation have been shown to increase inter-observer reliability (Guercio & Hahs, 2015).
- There may be item-level bias within the ADOS-2 for ethnicity and race (Harrison et al., 2017).
- ADOS-2 calibrated severity scores (CSS) were developed to provide a metric that is relatively independent of participants characteristics and can be used to compare the symptom severity of individuals with ASD across time (Janvier et al., 2022). One study found strong test re-test reliability of the CSS across all ADOS modules supporting their use as a reliable tool to quantify autism symptom severity across development (Janvier et al., 2022).

What this means in practice:

- ADOS-2 scores should be considered alongside all other information discovered during the assessment process, and through discussion with other involved people.
- Whilst studies suggest ADOS-2 scores below 7 indicate a person is not autistic, clinicians should also consider the possibility that a person is masking autistic features.

© NAIT and the Scottish ADOS Consortium 2022

- The CAT-Q assessment tool may be useful if you suspect this may be the case.
- ADOS assessors should regularly undertake calibration activities to ensure they are scoring reliably. This could include videoing assessment for discussion with a colleague or attending consensus coding workshops. It is helpful to check calibration with people outside the team you normally work with to make sure that you have not developed local anomalies in scoring.

8. How do you report findings?

- Be aware of who will read the report.
- Be aware of the language you use. Think 'difference, not deficit'.
- Focus on writing a detailed description of the individual's opportunities, actions and responses during the assessment rather than the final score.

Reports should include:

- Details of any deviation from advised administration of ADOS-2.
- Information concerning anything which may impact ADOS-2 scores including:
    - co-occurring conditions
    - communication
    - participants' state of mind
    - language preferences
- Any other information, which may contradict or corroborate the ADOS-2 score.
- The ADOS-2 score should not usually be included in reports as standard practice as it is easy for others reading the report to interpret this score in different ways (Lord et al 2012).

What this means in practice:

- Although ICD and DSM use deficit focussed language, this does not mean clinicians need to replicate this language. Autistic people usually prefer neurodiversity affirming language. Some examples of saying the same thing, but with positive language, are here.
- There is a sample report in the ADOS Manual pp. 221-223 (Lord et al., 2012).
- Including information from the ADOS assessment within the report of the overall diagnostic assessment is good practice.

9. Is ADOS compliant with a neurodiversity informed perspective?

- Receiving an autism diagnosis can be helpful for understanding, self-acceptance , positive identity and for accessing support services and reasonable adjustments (Arnold et al., 2020).
- ADOS-2 is framed in the current dominant deficit model described in diagnostic criteria rather than identifying strengths and preferences.
- Although not devised using a neurodiversity paradigm, the ADOS-2 might be one of the best ways of identifying autism in a clinical setting at the present time (Timimi et al., 2019).

What this means in practice:

- Assessors must be clear that the utility of making a diagnosis is not making any value judgement about the person.
- The assessor should inform the person about the nature and content of the assessment as part of the decision-making process about whether to undergo ADOS assessment.
- The ADOS-2 should not be used as the sole source of information in diagnostic assessment.
- Expert administration of ADOS is required to ensure the presses elicit social behaviours that may otherwise be hidden by masking, where this is appropriate. Expert administrators should also be aware of the risks of discouraging masking in environments, or with people who are unfamiliar. Feeling safe is the priority.
- Assessors can enhance practice by also understanding neurodiversity informed practice.

© NAIT and the Scottish ADOS Consortium 2022

10. Does ADOS work equally well for all genders and ethnic/ cultural backgrounds?

- There may be item level bias in ADOS for ethnicity and race (Harrison et al., 2017). Ethnic and racial minorities are underrepresented in autism research. Black children were more likely to have higher ratings on ADOS items assessing levels of unusual eye contact, stereotyped or idiosyncratic word use, and immediate echolalia.
- Research supports a male gender bias in ADOS scores (Adamou et al., 2018). One study found males had higher total CSS scores than female participants. Specifically, females were more likely to direct a range of facial expressions, and to identify and share emotions. There were also sex differences in offering information, reporting events, conversation, gestures, shared enjoyment, insight, amount of reciprocal social communication, quality of rapport, and imagination (Rea et al., 2022).

What this means in practice:

- Assessors should be aware of the risk of stereotyping and consider cultural differences in eye contact, facial expression, non-verbal cues and language and the possible impact they have upon ADOS scores.
- It is important to consider the impact on conversation and other verbal activities for people who do not have the same first language as the person conducting the assessment.

Further Reading

- Assessment, diagnosis and interventions for autism spectrum disorders (sign.ac.uk)
- Overview | Autism spectrum disorder in adults: diagnosis and management | Guidance | NICE
- https://www.beateatingdisorders.org.uk/get-information-and-support/about-eating-disorders/types/arfid/
- NAIT Guide to Autistic Masking

References

Adamou, M., Johnson, M., & Alty, B. (2018). Autism Diagnostic Observation Schedule (ADOS) scores in males and females diagnosed with autism: a naturalistic study. *Advances in Autism*, *4*(2), 49-55. https://doi.org/10.1108/AIA-01-2018-0003

Adamou, M., Jones, S. L., & Wetherhill, S. (2021). Predicting diagnostic outcome in adult autism spectrum disorder using the autism diagnostic observation schedule, second edition. *BMC Psychiatry*, *21*. https://doi.org/10.1186/s12888-020-03028-7

Arnold, S. R. C., Huang, Y., Hwang, Y. I. J., Richdale, A. L., Troller, J. N., & Lawson, L. P. (2020). "The Single Most Important Thing That Has Happened to Me in My Life": Development of the Impact of Diagnosis Scale - Preliminary Revisions. *Autism in Adulthood*, *2*(1). https://doi.org/10.1089/aut.2019.0059

Ashwood, K. L., Gillan, N., Horder, J., Hayward, H., Woodhouse, E., McEwen, F. S., . . . Murphy, D. G. (2016). Predicting the diagnosis of autism in adults using the Autism-Spectrum Quotient (AQ) questionnaire. *Psychological Medicine*, *46*(12), 2595-2604. https://doi.org/10.1017/S0033291716001082

Bal, V. H., Maye, M., Salzman, E., Huerta, M., Pepa, L., Risi, S., & Lord, C. (2020). The Adapted ADOS: A New Module Set for the Assessment of Minimally Verbal Adolescents and Adults. *Journal of Autism and Developmental Disorders*, *50*, 719-729. https://doi.org/https://doi.org/10.1007/s10803-019-04302-8

Bastiaansen, J. A., Meffert, H., Hein, S., Huizinga, P., Ketelaars, C., Pijnenborg, M., . . . de Bildt, A. (2011). Diagnosing autism spectrum disorders in adults: The use of Autism Diagnostic Observation Schedule (ADOS) module 4. *Journal of Autism and Developmental Disorders*, *41*(9), 1256-1266. https://doi.org/10.1007/s10803-010-1157-x

Brugha, T., Tyrer, F., Leaver, A., Lewis, S., Seaton, S., Morgan, Z., . . . Rensburg, K. (2020). Testing adults by questionnaire for social and communication disorders, including autism spectrum disorders, in an adult mental health service population. *International Journal of Methods in Psychiatric Research*, *29*(1). https://doi.org/10.1002/mpr.1814

Brugha, T. S., McManus, S., Smith, J., Scott, F. J., Meltzer, H., Purdon, S., . . . Bankart, J. (2012). Validating two survey methods for identifying cases of autism spectrum disorder among adults in the community. *Psychological Medicine*, *42*(3), 647-656. https://doi.org/10.1017/S0033291711001292

de Bildt, A., Sytema, S., Meffert, H., & Bastiaansen, J. A. C. J. (2016). The Autism Diagnostic Observation Schedule, module 4: Application of the revised algorithms in an independent, well-defined, Dutch sample (n = 93). *Journal of Autism and Developmental Disorders*, *46*(1), 21-30. https://doi.org/10.1007/s10803-015-2532-4

Fusar-Poli, L., Brondino, N., Rocchetti, M., Panisi, C., Provenzani, U., Damiani, S., & Politi, P. (2017). Diagnosing ASD in adults without ID: Accuracy of the ADOS-2 and the ADI-R. *Journal of Autism and Developmental Disorders*, *47*(11), 3370-3379. https://doi.org/10.1007/s10803-017-3258-2

Guercio, J. M., & Hahs, A. D. (2015). Applied behavior analysis and the Autism Diagnostic Observation Schedule (ADOS): A symbiotic relationship for advancements in services for individuals with autism spectrum disorders (ASDs). *Behavior Analysis in Practice*, *8*(1), 62-65. https://doi.org/10.1007/s40617-014-0034-z

Harrison, A. J., Long, K. A., Tommet, D. C., & Jones, R. N. (2017). Examining the role of race, ethnicity, and gender on social and behavioral ratings within the Autism Diagnostic Observation Schedule. *Journal of Autism and Developmental Disorders*, *47*(9), 2770-2782. https://doi.org/10.1007/s10803-017-3176-3

Hayashi, W., Hanawa, Y., Yuriko, I., Aoyagi, K., Saga, N., Nakamura, D., & Iwanami, A. (2022). ASD symptoms in adults with ADHD: A preliminary study using the ADOS-2. *European Archives of Psychiatry and Clinical Neuroscience*, *272*(2), 217-232. https://doi.org/10.1007/s00406-021-01250-2

Holzinger, D., Weber, C., Bölte, S., Fellinger, J., & Hofer, J. (2022). Assessment of autism spectrum disorder in deaf adults with intellectual disability: Feasibility and psychometric properties of an adapted version of the autism diagnostic observation schedule (ADOS-2). *Journal of Autism and Developmental Disorders*, *52*(7), 3214-3227. https://doi.org/10.1007/s10803-021-05203-5

Hus, V., & Lord, C. (2014). The Autism Diagnostic Observation Schedule, Module 4: Revised algorithm and standardized severity scores. *Journal of Autism and Developmental Disorders*, *44*(8), 1996-2012. https://doi.org/10.1007/s10803-014-2080-3

Janvier, D., Choi, Y. B., Klein, C., Lord, C., & Kim, S. H. (2022). Brief report: Examining test-retest reliability of the Autism Diagnostic Observation Schedule (ADOS-2) calibrated severity scores (CSS). *Journal of Autism and Developmental Disorders*, *52*(3), 1388-1394. https://doi.org/10.1007/s10803-021-04952-7

Kamp-Becker, I., Albertowski, K., Becker, J., Ghahreman, M., Langmann, A., Mingebach, T., . . . Stroth, S. (2018). Diagnostic accuracy of the ADOS and ADOS-2 in clinical practice. *European Child & Adolescent Psychiatry*, *27*(9), 1193-1207. https://doi.org/10.1007/s00787-018-1143-y

Kästner, A., Begemann, M., Michel, T. M., Everts, S., Stepniak, B., Bach, C., . . . Ehrenreich, H. (2015). Autism beyond diagnostic categories: Characterization of autistic phenotypes in schizophrenia. *BMC Psychiatry*, *15*. https://doi.org/10.1186/s12888-015-0494-x

Langmann, A., Becker, J., Poustka, L., Becker, K., & Kamp-Becker, I. (2017). Diagnostic utility of the autism diagnostic observation schedule in a clinical sample of adolescents and adults. *Research in Autism Spectrum Disorders*, *34*, 34-43. https://doi.org/10.1016/j.rasd.2016.11.012

Lord, C., Rutter, M., DiLavore, P. C., Risi, S., Gotham, K., & Bishop, S. (2012). *Autism Diagnostic Observation Schedule, Second Edition (ADOS-2) [Manual: Modules 1-4]* (Second ed.). Western Psychological Sefvices.

Maddox, B. B., Brodkin, E. S., Calkins, M. E., Shea, K., Mullan, K., Hostager, J., . . . Miller, J. S. (2017). The Accuracy of the ADOS-2 in Identifying Autism among Adults with Complex Psychiatric Conditions. *Journal of Autism and Developmental Disorders*, *47*(9), 2703-2709. https://doi.org/10.1007/s10803-017-3188-z

Medda, J. E., Cholemkery, H., & Freitag, C. M. (2019). Sensitivity and specificity of the ADOS-2 algorithm in a large German sample. *Journal of Autism and Developmental Disorders*, *49*(2), 750-761. https://doi.org/10.1007/s10803-018-3750-3

Rea, H. M., Øien, R. A., Shic, F., Webb, S. J., & Ratto, A. B. (2022). Sex Differences on the ADOS-2. *Journal of Autism and Developmental Disorders*. https://doi.org/10.1007/s10803-022-05566-3

Rutherford, M., McKenzie, K., McClure, I., Forsyth, K., O'Hare, A., McCartney, D., & Finlayson, I. (2016). A national study to investigate the clinical use of standardised instruments in autism spectrum disorder assessment of children and adults in Scotland. *Research in Autism Spectrum Disorders*, *29-30*, 93-100. https://doi.org/10.1016/j.rasd.2016.05.003

Rydzewska, E., Hughes-Mccormack, L. A., Gillberg, C., Henderson, A., Macintyre, C., Rintoul, J., & Cooper, S.-A. (2018). Prevalence of long-term health conditions in adults with autism: observational study of a whole country population. *BMJ Open*, *8*(8), e023945. https://doi.org/10.1136/bmjopen-2018-023945

Sappok, T., Diefenbacher, A., Budczies, J., Schade, C., Grubich, C., Bergmann, T., . . . Dziobek, I. (2013). Diagnosing autism in a clinical sample of adults with intellectual disabilities: How useful are the ADOS and the ADI-R? *Research in Developmental Disabilities*, *34*(5), 1642-1655. https://doi.org/10.1016/j.ridd.2013.01.028

Schutte, J. L., McCue, M. P., Parmanto, B., McGonigle, J., Handen, B., Lewis, A., . . . Saptono, A. (2015). Usability and reliability of a remotely administered adult autism assessment, the Autism Diagnostic Observation Schedule (ADOS) Module 4. *Telemedicine and e-Health*, *21*(3), 176-184. https://doi.org/10.1089/tmj.2014.0011

Sedgewick, F., Kerr-Gaffney, J., Leppanen, J., & Tchanturia, K. (2019). Anorexia nervosa, autism, and the ADOS: How appropriate is the new algorithm in identifying cases? *Frontiers in Psychiatry*, *10*. https://doi.org/10.3389/fpsyt.2019.00507

Stadnick, N., Brookman-Frazee, L., Nguyen Williams, K., Cerda, G., & Akshoomoff, N. (2015). A pilot study examining the use of the autism diagnostic observation schedule in community-based mental health clinics. *Research in Autism Spectrum Disorders*, *20*, 39-46. https://doi.org/10.1016/j.rasd.2015.08.007

Taylor, L. J., Eapen, V., Maybery, M., Midford, S., Paynter, J., Quarmby, L., . . . Whitehouse, A. J. O. (2017). Brief report: An exploratory study of the diagnostic reliability for autism spectrum disorder. *Journal of Autism and Developmental Disorders*, *47*(5), 1551-1558. https://doi.org/10.1007/s10803-017-3054-z

Timimi, S., Milton, D., Bovell, V., Kapp, S., & Russell, G. (2019). Deconstructing Diagnosis: Four Commentaries on a Diagnostic Tool to Assess Individuals for Autism Spectrum Disorders. *Autonomy, the Critical Journal of Autism Studies*, *1*(6).

Trevisan, D. A., Foss-Feig, J. H., Naples, A. J., Srihari, V., Anticevic, A., & McPartland, J. C. (2020). Autism Spectrum Disorder and Schizophrenia are better Differentiated by Positive Sympstoms Than Negative Symptoms. *Frontiers in Psychiatry*, *11*(548). https://doi.org/10.3389/fpsyt.2020.00548

Westwood, H., & Tchanturia, K. (2017). Autism Spectrum Disorder in Anorexia Nervosa: An Updated Literature Review. *Current psychiatry reports*, *19*(7), 41. https://doi.org/10.1007/s11920-017-0791-9

Zheng, Z., Zheng, P., & Zou, X. (2018). Association between schizophrenia and autism spectrum disorder: A systematic review and meta-analysis. *Autism Research*, *11*(8), 1110-1119. https://doi.org/10.1002/aur.1977

© NAIT and the Scottish ADOS Consortium 2022
